# Factors associated with health-related quality of life among people living with HIV in South Korea: Tobit regression analysis

**DOI:** 10.1371/journal.pone.0303568

**Published:** 2024-05-16

**Authors:** Gwang Suk Kim, Layoung Kim, SangA Lee, Mi-So Shim, Youngjin Lee, Seoyoung Baek

**Affiliations:** 1 Mo-Im Kim Nursing Research Institute, College of Nursing, Yonsei University, Seoul, Republic of Korea; 2 College of Nursing and Brain Korea 21 FOUR Project, Yonsei University, Seoul, Republic of Korea; 3 College of Nursing, Yonsei University, Seoul, Republic of Korea; 4 Manning College of Nursing and Health Sciences, University of Massachusetts, Boston, Massachusetts, United States of America; 5 College of Nursing, Keimyung University, Daegu, Republic of Korea; Iowa State University, UNITED STATES

## Abstract

This study investigated health-related quality of life and identified factors affecting it among people with the HIV in South Korea. A total of 243 people living with HIV participated in this cross-sectional survey. Data were collected from five hospitals between November 2021 and August 2022 using structured online questionnaires. Data were analyzed using descriptive statistics, Mann-Whitney U test, Kruskal-Wallis test, Spearman’s rho analysis, and Tobit regression analysis because a significant ceiling effect was observed for the dependent variable. The mean score for the health-related quality of life was 75.74 ± 16.48. The significant factors that positively influence the health-related quality of life were “employment” (B = 4.57, *p* = .035), “not participating in the self-help group” (B = 6.10, *p* = .004), “higher self-efficacy for managing symptoms” (B = 1.32, *p* = .036), “higher self-efficacy for getting support/help” (B = 0.95, *p* = .035), and “higher self-efficacy for managing fatigue” (B = 2.80, *p* < .001) in the Tobit regression analysis. The results suggest that interventions to increase self-efficacy should involve developing programs and policies for people living with HIV. There is a need for efforts to provide healthcare services linked to employment support, as well as to establish a social environment in which they can work without stigma. Further, self-help groups could be utilized as intervention channels.

## Introduction

As of 2020, there were an estimated 37 million people living with HIV (PLWH) worldwide. Despite the various efforts aimed at ending the HIV/AIDS epidemic by 2030 (UN General Assembly), the number of new HIV infections reached 1.5 million in 2021 [1]. Even with the rate of new infections declining, the number of PLWH is expected to increase as the life expectancy approaches that of the general population owing to increased access to treatment. As of 2021, there are approximately 15,000 PLWH in South Korea [2]; and their drug treatment rate was 95.5% and the viral suppression rate was 96.0%, exceeding the UNAIDS “90-90-90 target” (90% of PLWH know their HIV status, 90% of PLWH receive sustained antiretroviral therapy, and 90% of people receiving antiretroviral therapy have viral suppression) [1–3]. Although achieving the global goals, Kim et al. [4] reported that only 54% of PLWH disclosed their HIV status to an important supporter, and participation in self-help groups was low at 15%. This is related to cultural and social factors that make it difficult to accept HIV diagnosis from family and others [5].

The widespread use of effective antiretroviral therapy has transformed HIV into a chronic disease [6]. There has been increased interest in the quality of life (QoL) of PLWH among researchers and healthcare providers [7]. For PLWH, QoL is defined as the “fourth 90” after achieving the “90-90-90 target,” which implies that 90% of PLWH who remain virally suppressed will have a good health-related QoL (HRQoL) [8]. While it is often assumed that increased adherence to treatment among PLWH leads to improved QoL, even in virally suppressed states, QoL among PLWH remains low compared with that of the general population [9]. This highlights that viral suppression is not the ultimate treatment goal for PLWH and that QoL must be addressed with attention to mental health, chronic disease, and pain management, as well as social stigma and discrimination, to improve health outcomes [10].

The World Health Organization defines QoL as “an individual’s perception of his or her place in life in relation to his or her goals, expectations, standards, and interests in the context of the culture and value system in which he or she lives” [11], demonstrating that understanding an individual’s QoL includes not only individual but also sociocultural factors. Domestic and international studies have identified higher self-efficacy, higher social support, psychological well-being, sex (male), younger age, higher economic level (monthly household income), and employment status (employed) as factors that positively influence HRQoL among PLWH [12–16]; and social stigma, stress, and low treatment adherence as factors that negatively affect QoL [15–18], demonstrating that various personal and sociocultural factors affect the QoL of PLWH. Although previous studies have identified factors affecting QoL among PLWH in South Korea [15,16], they could be outdated or cover only a few independent variables, which may not reflect the characteristics of newly infected people and the various personal and sociocultural characteristics of PLWH. Among these various factors, the factors that can be changed and improved need to be established. To achieve a healthy life beyond viral suppression, it is important to focus on factors that can be improved and how they can be approached. Therefore, this study aimed to identify the factors that affect the HRQoL of PLWH in a multifaceted manner.

## Materials and methods

### Study design

This descriptive study aimed to identify the factors affecting the HRQoL of PLWH through a secondary data analysis. This study was conducted using data from participants of a parent study [19], which evaluated the validity and reliability of self-efficacy for HIV disease management skills among South Korean participants.

### Sample

The inclusion criteria for participating in the parent study were PLWH aged 19 years or older who had been diagnosed more than one month prior to the participation. PLWH were recruited using probabilistic convenience sampling from five hospitals conducting a counseling project organized by the Korean Disease Control and Prevention Agency. A total of 243 PLWH participated in this study from November 18, 2021, to August 15, 2022, and all these data were used in our study. The online survey system was designed so that all items had to be responded to before participants could proceed to the next page and complete the survey. There were no missing data.

We used the G*power 3.1.9.2 to calculate a significance level of.05, a moderate effect size of.15, a sample size of 243, and 13 independent variables included in the regression model. The results showed a power of 98.9%, confirming that the sample size was sufficient for determining the influence of the dependent variable and to achieve the study purpose.

### Data collection

PLWH visiting the outpatient departments of infectious diseases at the five hospitals were provided with a participant recruitment guide specifying the purpose, method, and inclusion criteria of this study, and an online survey URL. Participants were asked to voluntarily participate in the online survey.

### Variables and measures

#### HRQoL

HRQoL, the dependent variable in this study, was measured using the EuroQoL group’s visual analogue scale (VAS), a common health measurement tool [20]. The EQ-VAS measures a person’s perceived health on a particular day on a scale ranging from the *worst health imaginable* (0) to the *best health imaginable* (100), with higher scores indicating a higher subjective HRQoL. The EQ-VAS, along with the subdomain-specific items of the EQ-5D, have been used to measure HRQoL in various populations and in studies on PLWH [21,22]. We used a single item, the EQ-VAS, to determine the HRQoL of participants, which was easy to answer and allowed us to measure their general health status as a ratio variable.

#### Self-efficacy for HIV disease management

Self-efficacy for HIV disease management among PLWH was measured using an instrument developed by Shively et al. [23] and adapted by Kim et al. [19]. It comprises 33 items and 6 domains: “managing depression/mood” (9 items), “managing medications” (6 items), “managing symptoms” (5 items), “communicating with healthcare provider” (4 items), “getting support/help” (5 items), and “managing fatigue” (4 items), which were rated on a 10-point Likert scale (1 *not at all* to 10 *very much*). Higher scores indicate higher self-efficacy in managing HIV. Cronbach’s alpha for the study of Shively et al. [23] was.96 that for the Korean instrument [19] was.91. In the previous studies, six domains were analyzed and used to identify specific concepts of self-care efficacy [24–26].

#### Medication adherence

Medication adherence was measured using a self-report measure for self-checking the percentage of medication adherence in the past month, which was proposed by Simoni et al. [27] and adapted to South Korean conditions for the evaluation of a healthcare counseling project. The scores ranged from 0 to 100, with 0% indicating no prescribed medication and 100% indicating that all prescribed medications were taken.

#### Disease-related characteristics

Disease-related characteristics included seven questions about the duration of HIV diagnosis, place of diagnosis, duration from diagnosis to treatment initiation, self-help group participation, current care stage, CD4 count, and the result of viral load test. The current care stage was divided into three phases: diagnosis to starting medication, starting medication to medication settlement, and medication settlement to sustaining health management.

#### General characteristics

General characteristics included eight questions on sex, year of birth, marital status, living status, education level, employment, economic status, and sexual identity. Regarding living status, those living alone were categorized as living alone and those living with family, friends, partners, or others were categorized as living together. Education level was categorized as high school or less for those who graduated from elementary, middle, or high school; and college or higher for those who graduated from college, university, or graduate school. For economic status, the question “What is your economic level?” was asked, and participants answered either high, medium, or low.

### Data analysis

The data of the parent study have been accessed for research purposes with being fully anonymized from February 26th to April 5th, 2023. The collected data were analyzed using STATA IC 16.1 (StataCorp, College Station, TX, USA). Participants’ general and disease-related characteristics, healthcare self-efficacy, medication adherence, and HRQoL were analyzed using descriptive statistics of real numbers and percentages, means, and standard deviations. The Mann-Whitney U test, Kruskal-Wallis test, and Spearman’s rho analysis were used to determine the differences in HRQoL based on participants’ general and disease-related characteristics, self-efficacy for health management, and medication adherence. Finally, Tobit regression analysis was conducted to identify factors affecting the HRQoL of PLWH. The dependent variable, HRQoL, had a negatively skewed distribution and ceiling effect (S1 Fig). Given the likelihood that we could obtain biased estimates from the ordinary least square method such as a linear regression model, we used Tobit regression analysis, which is designed to estimate linear relationships between variables when left or right censoring exists in the dependent variable [28].

S1 Fig showing the distribution of scores for the dependent variable, health-related quality of life. On a scale of 0 to 100, the minimum response is 15 and the maximum is 100. The mean of the scores is 75.74, with a median of 80.00.

### Ethical considerations

This study, using secondary data, was approved for exemption from deliberation by the Institutional Review Board to which the researchers belong (no. 4-2022-1660). There was no additional data collection process. It was explained to participants that the data would not be used for anything other than statistical and academic research purposes and would be kept confidential. This study has utilized data from participants of the parent study who understood the study purpose and agreed to the use of their data for academic research purposes in the future. The requirement for written informed consent was waived in the parent study because participants were considered to have agreed to participate in the study only if they clicked on the online survey link and checked the box to agree to participate in the online survey. The data of the parent study were fully anonymized without any personal information (names, social security numbers, mobile phone numbers, etc.) and contained no information that could be used to infer who participants were.

## Results

### Demographic and disease related characteristics of participants

Most (92%) participants were male. The average values of age and time length of HIV diagnosis were 38.65 ± 9.92 (range: 21–73 years) and 8.58 ± 7.10 years, respectively. Approximately 30% of the participants were unemployed (31.7%) and the number of people who participated in self-help groups was 29.2%. The mean score for self-efficacy for HIV disease management and HRQoL were 6.83 ± 1.32 out of 10 and 75.74 ± 16.48 out of 100, respectively (Table 1).

**Table 1:** Differences in health-related quality of life based on demographics, disease-related characteristics, and self-efficacy (*N* = 243).

Variables	Categories	n (%)	Mean ± SD	Health-related quality of life	
				Median (IQR)	U, H, *ρ* (*p*)
Demographic characteristics					
Sex	Male	229 (94.2)		80.0 (19.0)	1618.5 (.951)
	Female	14 (5.8)		80.0 (25.0)	
Age (years)			38.65 ± 9.92		-.20 (.002)
Marital status	Single	221 (90.9)		80.0 (20.0)	6.61 (.085)
	Married	4 (1.6)		82.5 (10.0)	
	Divorce	15 (6.2)		68.0 (30.0)	
	Bereavement	3 (1.2)		70.0 (22.5)	
Living status	Living alone	141 (58.0)		80.0 (16.0)	6054.5 (.034)
	Living together	102 (42.0)		80.0 (20.0)	
Education level	≤ High school	91 (37.4)		70.0 (25.0)	5073.5 (< .001)
	≥ College, university	152 (62.6)		80.0 (20.0)	
Employment	Unemployed	77 (31.7)		70.0 (30.0)	4259.5 (< .001)
	Employed	166 (68.3)		80.0 (20.0)	
Economic status	High	10 (4.1)		85.0 (20.0)	17.65 (< .001)
	Middle	110 (45.3)		80.0 (20.0)	
	Low	123 (50.6)		75.0 (20.5)	
Sexual identity	Homosexual	165 (67.9)		80.0 (20.0)	1.46 (.691)
	Heterosexual	35 (14.4)		80.0 (32.5)	
	Bisexual	38 (15.6)		80.0 (15.0)	
	Others	5 (2.1)		70.0 (30.0)	
Disease-related characteristics					
Time length of HIV diagnosis (months)			103.58 ± 85.22		-.14 (.031)
Place of diagnosis	Hospital	134 (55.1)		80.0 (21.0)	4.53 (.210)
	Public health center	80 (32.9)		80.0 (16.0)	
	Military manpower administration	14 (5.8)		86.5 (10.0)	
	Others	15 (6.2)		80.0 (12.5)	
Duration from diagnosis to treatment start (months)			11.33 ± 33.54		-.06 (.369)
Self-help group participation	Yes	71 (29.2)		75.0 (15.5)	5024.0 (.029)
	No	172 (70.8)		80.0 (20.0)	
Care stage	Diagnosis ~ starting medication	9 (3.7)		75.0 (14.0)	2.51 (.285)
	Starting medication ~ medication settlement	19 (7.8)		75.0 (39.0)	
	Medication settlement ~ sustaining health management	215 (88.5)		80.0 (20.0)	
CD4 count	< 200 cells/mm^3^	6 (2.5)		79.0 (31.0)	0.18 (.916)
	≥ 200 cells/mm^3^	173 (71.2)		80.0 (15.0)	
	Don’t know	64 (26.3)		80.0 (22.0)	
Viral load test	Detected	6 (2.5)		85.0 (25.0)	1.87 (.392)
	Undetected	200 (82.3)		80.0 (18.0)	
	Don’t know	37 (15.2)		75.0 (27.0)	
Self-efficacy for HIV disease management (1–10)			6.83 ± 1.32		.52 (< .001)
Managing depression / mood			6.15 ± 1.74		0.41 (< .001)
Managing medications			8.79 ± 1.19		0.25 (< .001)
Managing symptoms			7.44 ± 1.86		0.46 (< .001)
Communicating with healthcare provider			7.77 ± 2.31		0.31 (< .001)
Getting support / help			4.31 ± 2.32		0.27 (< .001)
Managing fatigue			6.68 ± 2.14		0.55 (< .001)
Medication adherence (0–100)			96.72 ± 6.42		-.01 (.819)

SD, standard deviations; IQR, inter quartile range; U, U statistic of Mann-Whitney U test; H, H statistic of Kruskal-Wallis test; ***ρ***, Sperman’s rho; *p*, p-value.

### HRQoL according to characteristics

The HRQoL was positively correlated with self-efficacy (*ρ* = .52, *p* < .001) and negatively correlated with age (*ρ* = -.20, *p* = .002), time length of HIV diagnosis (*ρ* = -.14, *p* = .031). There were differences in HRQoL according to living status (U = 6054.5, *p* = .034), education level (U = 5073.5, *p* < .001), employment (U = 4259.5, *p* < .001), economic status (H = 17.65, *p* < .001), and self-help group participation (U = -5024.0, *p* = .029; Table 1). Those living together, college graduates and above, those who were employed, and those who did not participate in the self-help group had higher levels of HRQoL than those living alone, high school graduates and below, those who were unemployed, and those who participated in the self-help group, respectively.

### Factors associated with HRQoL

**Fig 1:**
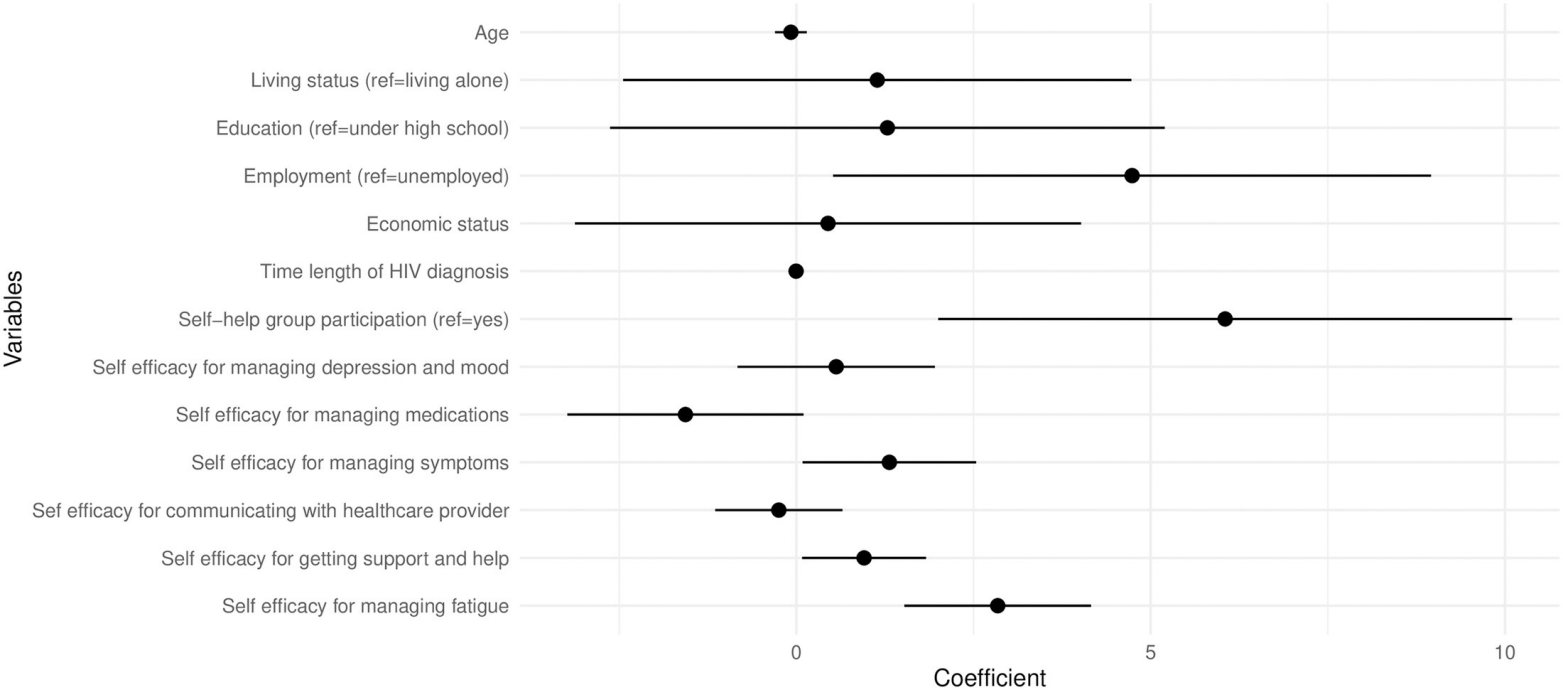
Forest plot showing the tobit regression coefficient. Forest plot showing coefficient and associated 95% confidence interval (CI) for variables that affect health related quality of life in the Tobit regression.

The results of the Tobit regression analysis are shown in Table 2. Employed participants had in average 4.57 higher scores than those unemployed. Those who did not participate in the self-help group had a 6.10 higher HRQoL score than those who did. Higher scores for self-efficacy for managing symptoms (B = 1.32, *p* = .036), self-efficacy for getting support/help (B = 0.95, *p* = .035), and self-efficacy for managing fatigue (B = 2.80, *p* < .001) were associated with higher HRQoL scores (Table 2, Fig 1).

**Table 2:** Factors affecting health-related quality of life (*N* = 243).

Variables	Coefficient	SE	t (*p*)	95% CI	
Age	-0.09	0.12	0.79 (.430)	-0.32	0.14
Living status (ref = living alone)	1.01	1.84	0.55 (.582)	-2.61	4.63
Education (ref = under high school)	1.17	2.00	0.59 (.558)	-2.77	5.11
Employment (ref = unemployed)	4.57	2.16	2.12 (.035)	0.31	8.83
Economic status	0.51	1.82	0.28 (.781)	-3.09	4.10
Time length of HIV diagnosis	-0.01	0.01	0.34 (.732)	-0.03	0.02
Self-help group participation (ref = yes)	6.10	2.07	2.95 (.004)	2.02	10.17
Self-efficacy for HIV disease management					
Managing depression/mood	0.66	0.72	0.92 (.361)	-0.76	2.07
Managing medications	-1.53	0.85	1.80 (.074)	-3.21	0.15
Managing symptoms	1.32	0.63	2.11 (.036)	0.09	2.55
Communicating with healthcare provider	-0.25	0.46	0.55 (.585)	-1.15	0.65
Getting support/help	0.95	0.45	2.12 (.035)	0.07	1.83
Managing fatigue	2.80	0.67	4.15 (< .001)	1.47	4.13
LR *x*^2^	116.10				
Prob > *x*^2^	< .001				

SE, standard error; CI, confidence intervals; t, an indicator used to evaluate the statistical significance of a coefficient; *p*, p-value; ref, reference; LR, likelihood ratio; Prob > *x*^2^, probability of getting a LR test statistic as extreme as, or more so, than the observed statistic under the null hypothesis.

## Discussion

This study was conducted to identify the HRQoL among PLWH and the factors that influence it. We conducted a Tobit regression analysis reflecting the characteristics of the dependent variable. Employment, not participating in the self-help group, higher self-efficacy for managing symptoms, higher self-efficacy for getting support/help, and higher self-efficacy for managing fatigue were identified as factors positively associated with HRQoL among PLWH. The results are meaningful in that they provide a basis for interventions to improve the HRQoL of PLWH.

A previous study, based in the UK, reported that HRQoL of PLWH is still low compared to that of the general population [9], but it is higher than it used to be as single-tablet regimens are developed and adherence to medication increases [6]. The HRQoL was 75.74, with most of the scores in the high range, indicating that the HRQoL of PLWH was high. To reflect the fact that the HRQoL of PLWH has increased, this study used Tobit regression analysis to explore the factors affecting HRQoL. Tobit regression analysis could be used when the distribution of dependent variable showed skewed and ceiling effect [28]. A previous study comparing multiple, logit, and Tobit models showed that the Tobit regression analysis is more appropriate than multiple linear regression analysis when the dependent variable has a floor effect, emphasizing the need to use the appropriate analysis method for the research topic and context [29]. Consequently, a Tobit regression analysis has been used in studies exploring HRQoL determinants in other populations as well as among PLWH [30–32]. It is reasonable to check the distribution of HRQoL, which is the collected research data, and to use a Tobit regression analysis. Nevertheless, the authors were concerned about the possibility of a skewed distribution owing to convenience sampling [33], which may have increased the proportion of participants with above average HRQoL compared to HRQoL in the population. Therefore, it is recommended to plan a sampling method that is more representative of the population in advance.

The Tobit regression analysis findings indicated that those who had a job had higher HRQoL than those who were unemployed. It is common for unemployment to lead to low HRQoL. However, with South Korea’s unemployment rate among general population aged 15 years or older at 2.9% as of 2022 [34], it is noteworthy that the unemployment rate was 31.7%, despite the fact that the average age of the participants in this study was approximately 39. PLWH face several barriers to employment, including the physical symptoms of HIV and side effects of HIV medication, mental health challenges, fewer job options, fear of discrimination, and fear of loss of benefits, such as health insurance [35]. Contrastingly, social support, work environment, and work schedules are factors that facilitate their employment [35]. As the life expectancy of PLWH continues to increase, providing them with jobs is crucial to their social independence [35]. Moreover, employment status is a critical factor in the transmission of HIV infection as it is positively associated with HIV testing, linkage to HIV care, retention in HIV care, and HIV medication adherence [35]. Therefore, there is a need for efforts to provide healthcare services linked to employment support, as well as to establish a social environment in which they can work without stigma.

Those who did not participate in self-help groups had a higher QoL than those who did. These results lead to consider the two variables in terms of association, not causality. PLWH with low HRQoL are likely to participate in self-help groups as a way of seeking help, meanwhile PLWH with high HRQoL are more likely to believe that they do not need to participate in self-help groups. Self-help group participants among PLWH are more likely to be unemployed, have low incomes, and have low education levels [36,37]. In this study, the employment rate for those who did not participate in the self-help groups was 70.9%, higher than the 62.0% rate for those who did not participate. Considering that PLWH participate in self-help groups to be part of a social network, find useful information, and receive emotional support [38,39], the self-help groups could be considered as intervention channels. Furthermore, in this study, self-efficacy in obtaining support/help of those who participated in the self-help group (5.37 out of 10) was stronger than those who did not participate (3.87 out of 10), and given that self-efficacy in obtaining support/help was significant affecting factor of HRQoL, self-help groups may be a strategy to increase HRQoL in low socioeconomic status individuals. Given that PLWH are often reluctant to disclose their infection and could intentionally seek resources in distant communities or prefer online communities [36], utilizing anonymous online communities to form self-help groups could be a viable strategy.

Finally, self-efficacy in managing symptoms, obtaining support/help, and managing fatigue was associated with a good HRQoL of PLWH. Previous studies using the same self-efficacy measurement as this study also reported positive correlations between QoL of PLWH and self-efficacy for managing symptoms, getting support/help, and managing fatigue [24], confirming that self-efficacy is a significant predictor of QoL [25]. Self-efficacy was associated with better QoL in social relationships and physical and psychological independence, and overall social environment domain [40], suggesting that self-efficacy can directly influence health promotion behavior. PLWH could experience a range of symptoms owing to HIV infection, medication side effects, and medical complications [41], and fatigue are a pervasive symptom among PLWH [42]. Thus, it is important to help them recognize that they may experience symptoms, render them capable of managing them, and reassure them that they have the ability to seek help when needed. In addition, interventions to increase self-efficacy, focusing on symptom management and help-seeking, may not only improve overall QoL but also help them gain the strength to carry on with daily life. In particular, although self-efficacy for obtaining support/help was identified as a predictor of HRQoL in this study, the participants’ score for obtaining self-efficacy for obtaining support/help was low (4.31 out of 10). PLWH are often reluctant to seek help from others owing to fear of social stigma; however, given the mediating effect of self-efficacy on social stigma and QoL, interventions to increase self-efficacy in getting support/help are important [43].

There was a similar sex distribution to that of previous studies that utilized data from the National Health Insurance and Korea HIV/AIDS cohort studies [44,45]. However, the average age of the participants (38.65 ± 9.92) in this study was lower than the average age of the participants in the other study (41.5 ± 12.5), and a higher proportion of single individuals was identified in terms of marital status [45]. Considering that the previous studies used data before 2016, it is unclear whether this difference is owing to changes in the distribution of participants over time or owing to the sampling method of this study. Despite that data were collected at five hospitals while monitoring the characteristics of participants, it is necessary to compare the results with other studies in the future.

This study has some limitations. This was a secondary data analysis of a parent study that used convenience sampling. Given that the Korean HIV/AIDS cohort covers 16 hospitals and about 1000 PLWH [46], the number of participants and the number of institutions that collected data are not small. Although the parent study recruited participants by monitoring their general characteristics in five hospitals to maximize the representativeness of the sample, there are limitations in the sample being fully representative of the population because participants were recruited from five tertiary hospitals located in a metropolitan city. It is possible that the convenience sampling contributed to the high HRQoL and skewness of the dependent variable. There are currently limited studies reporting HRQoL among PLWH in South Korea; however, if further studies are reported, they could be compared with the current results. Moreover, this study was a secondary analysis of existing data, and it was limited by the fact that it did not include stigma and comorbidity, which are factors that can affect HRQoL. It could also be a limitation that self-reported questionnaires were used to get information about CD4 count and viral load test results. The number of “don’t know” responses was high, and the contents of the responses could be inaccurate; thus, in the future, it could be a good alternative to utilize real data in conjunction with electronic records. In addition, this was a cross-sectional study; therefore, the identification of causal relationships between independent variables and HRQoL is limited.

## Conclusions

This study identified the factors that influence HRQoL among PLWH using a Tobit regression analysis, which reflects the distribution of the dependent variable. Employment, self-help group participation, and self-efficacy influenced the HRQoL of PLWH. Our findings highlight the need for policies and social culture to ensure employment, and practical intervention to increase self-efficacy capabilities in symptom management and getting support to improve PLWHs’ HRQoL. A comprehensive approach linking healthcare service and employment welfare, and creating an inclusive culture of society is required.

## Supporting information

S1 Fig Distribution of dependent variable (health-related quality of life). (TIF)

S1 Table Multiple linear regression. (TIF)

S1 File (PDF)
