# Supplementary figures and images for: Factors associated with health-related quality of life among people living with HIV in South Korea: Tobit regression analysis
Source: PLoS One. 2024 May 16;19(5):e0303568. doi: 10.1371/journal.pone.0303568 (PMC11098325; doi:10.1371/journal.pone.0303568)

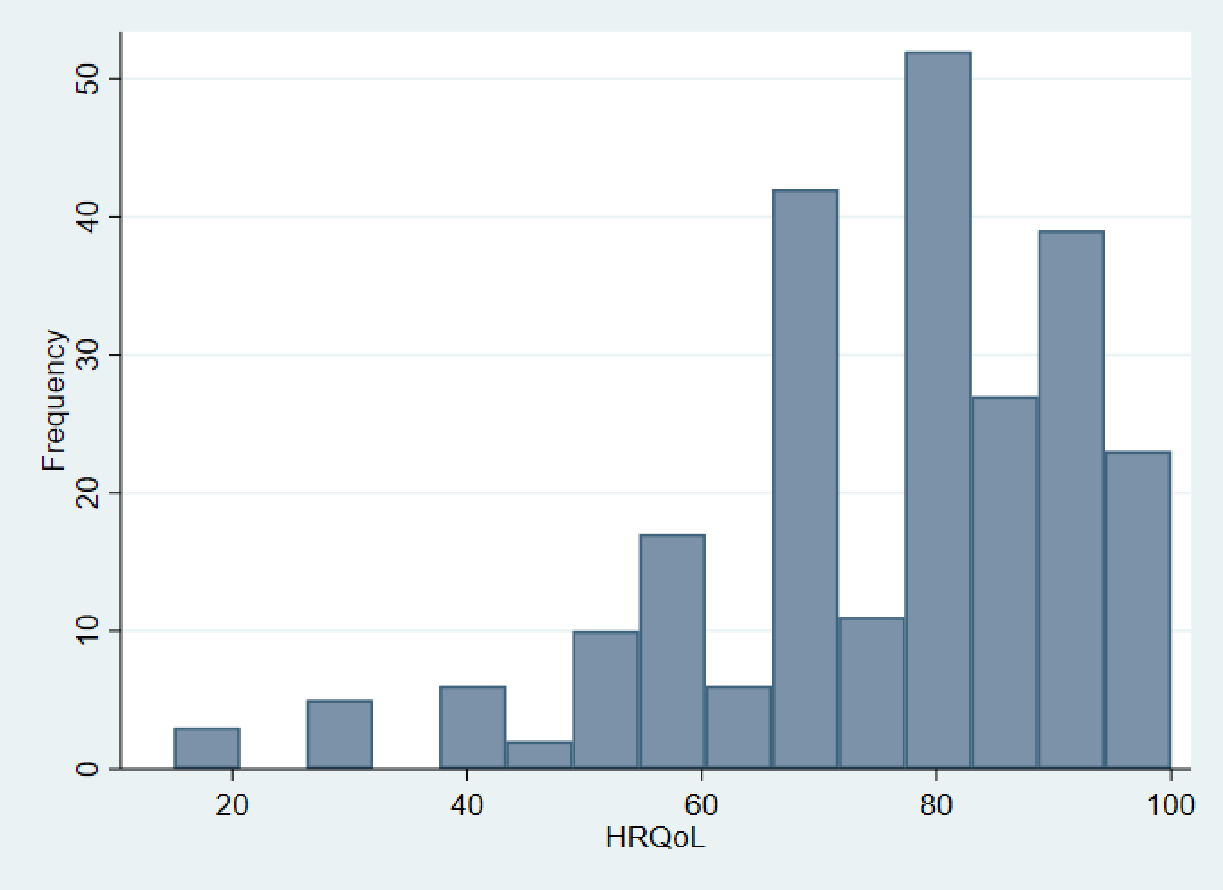

Supplement: S1 Fig — (TIF) [file pone.0303568.s001.tif]

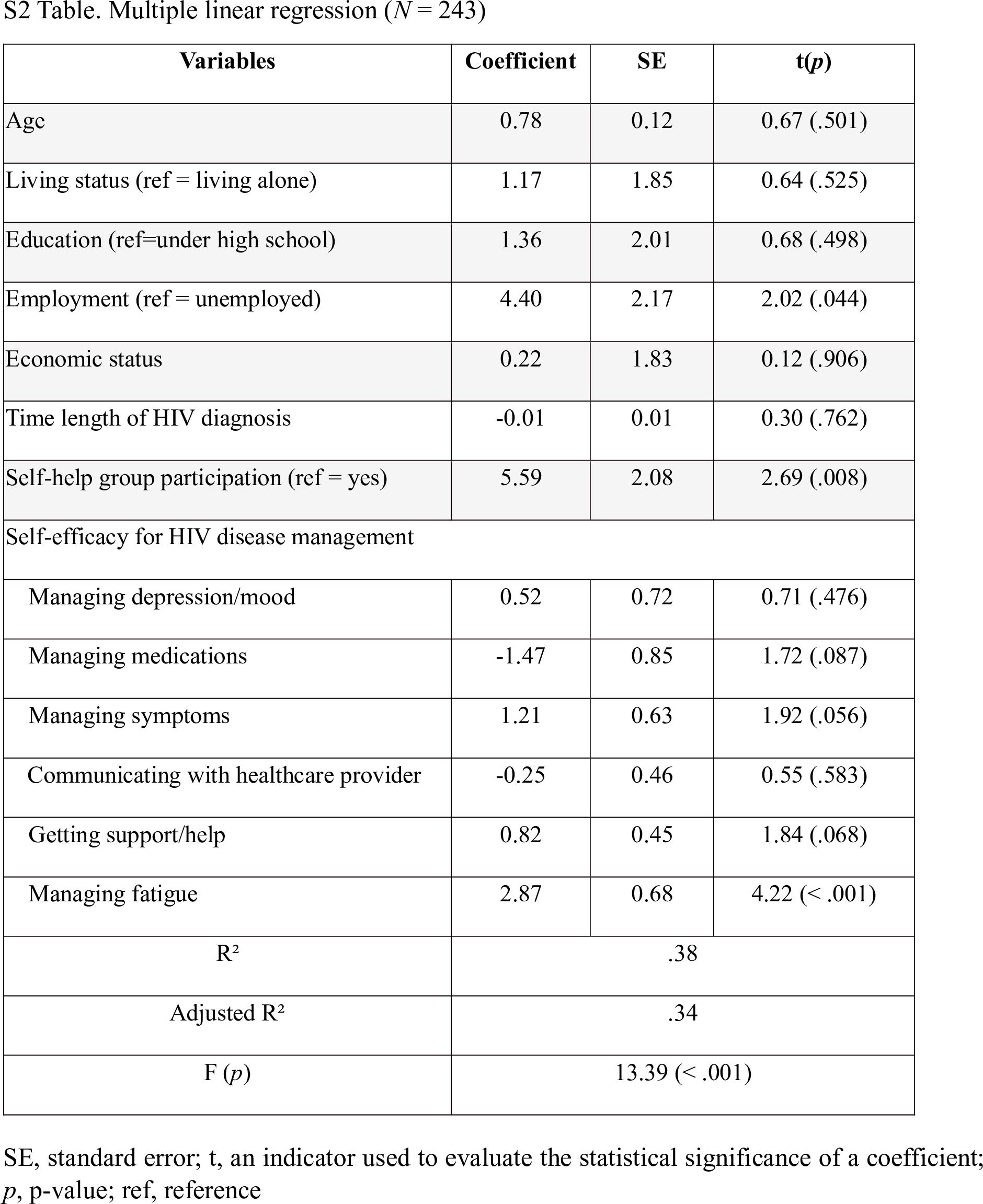

Supplement: S1 Table — (TIF) [file pone.0303568.s002.tif]
