# Supplementary material for: Factors associated with health-related quality of life among people living with HIV in South Korea: Tobit regression analysis
Source: PLoS One. 2024 May 16;19(5):e0303568. doi: 10.1371/journal.pone.0303568 (PMC11098325; doi:10.1371/journal.pone.0303568)
Supplement: S1 File — (PDF) [file pone.0303568.s003.pdf]

Report of Institutional Review Board

Requester: PLOS ONE

This letter is to inform you of the results of your confidential.

Type of Review	■ Initial Review □ Response for Approved with Modification □ Deferred<br>□ Tabled □ Modification □ cancellation of protocol □ end of study report<br>□ final report □ interim report □ others		
IRB No.	4-2022-1660	Date of approval	February 25th, 2023
Title of Proposal	Factors Associated with Health Related Quality of Life among People Living with HIV in Korea		
	Protocol No. -	Version No.	-
Investigator	Principal Investigator: Gwang Suk Kim, RN, PhD. / Professor / Yonsei University College of Nursing, Seoul, Korea		
Generic name	-	Brand Name	-
Phase	□ Phase I □ Phase II □ Phase III □ Phase IV<br>□ Biological equivalence test ■ Others		
Proposed period of study	Approval date of IRB ~ 2024-02-24		
Contents of Review	**<u>* List of initial review (reviewed by E-IRB system)</u>**<br>1. Application Form<br>2. Study Protocol (KOR)<br>3. Case Report Form<br>4. CV for Principle Investigator		
Date of Review	February 25th, 2023		
Result of Review	■ Approved □ Approval with Modification □ Deferred □ tabled		
Comment	None		

Severance Hospital IRB is organized and operates according to ICH-GCP and the applicable laws and regulations

February 25th, 2023
Chairperson of Institutional Review Board
Severance Hospital
Yonsei University, College of Medicine
Seoul 03722, Korea
